# Clones and Clusters of Antimicrobial-Resistant *Klebsiella* From Southwestern Nigeria

**DOI:** 10.1093/cid/ciab769

**Published:** 2021-12-01

**Authors:** Ayorinde O Afolayan, Anderson O Oaikhena, Aaron O Aboderin, Olatunde F Olabisi, Adewale A Amupitan, Oyekola V Abiri, Veronica O Ogunleye, Erkison Ewomazino Odih, Abolaji T Adeyemo, Adeyemi T Adeyemo, Temitope O Obadare, Monica Abrudan, Silvia Argimón, Sophia David, Mihir Kekre, Anthony Underwood, Abiodun Egwuenu, Chikwe Ihekweazu, David M Aanensen, Iruka N Okeke, NIHR Global Health Research Unit on Genomic Surveillance of Antimicrobial Resistance, Khalil Abudahab, Harry Harste, Dawn Muddyman, Ben Taylor, Nicole Wheeler, Pilar Donado-Godoy, Johan Fabian Bernal, Alejandra Arevalo, Maria Fernanda Valencia, Erik C D Osma Castro, K L Ravikumar, Geetha Nagaraj, Varun Shamanna, Vandana Govindan, Akshata Prabhu, D Sravani, M R Shincy, Steffimole Rose, K N Ravishankar, Jolaade J Ajiboye, Celia Carlos, Marietta L Lagrada, Polle Krystle V Macaranas, Agnettah M Olorosa, June M Gayeta, Elmer M Herrera, Ali Molloy, John Stelling, Carolin Vegvari

**Affiliations:** 1 Global Health Research Unit on Genomic Surveillance of Antimicrobial Resistance, Department of Pharmaceutical Microbiology, Faculty of Pharmacy, University of Ibadan, Ibadan, Nigeria; 2 Department of Medical Microbiology and Parasitology, Obafemi Awolowo University Teaching Hospitals Complex, Ile-Ife, Nigeria; 3 Department of Medical Microbiology and Parasitology, University College Hospital, Ibadan, Nigeria; 4 Department of Medical Microbiology and Parasitology, Osun State University Teaching Hospital, Teaching Hospital, Osogbo, Nigeria; 5 Centre for Genomic Pathogen Surveillance, Big Data Institute, University of Oxford, Oxford, United Kingdom; 6 Wellcome Genome Campus, Hinxton, United Kingdom; 7 Nigeria Centre for Disease Control, Jabi, Abuja, Nigeria

**Keywords:** antimicrobial resistance, genomic surveillance, *Klebsiella*, Nigeria

## Abstract

**Background:**

*Klebsiella pneumoniae* is a World Health Organization high-priority antibiotic-resistant pathogen. However, little is known about *Klebsiella* lineages circulating in Nigeria.

**Methods:**

We performed whole-genome sequencing (WGS) of 141 *Klebsiella* isolated between 2016 and 2018 from clinical specimens at 3 antimicrobial-resistance (AMR) sentinel surveillance tertiary hospitals in southwestern Nigeria. We conducted in silico multilocus sequence typing; AMR gene, virulence gene, plasmid, and K and O loci profiling; as well as phylogenetic analyses, using publicly available tools and Nextflow pipelines.

**Results:**

Phylogenetic analysis revealed that the majority of the 134 *K. pneumoniae* and 5 *K. quasipneumoniae* isolates from Nigeria characterized are closely related to globally disseminated multidrug-resistant clones. Of the 39 *K. pneumoniae* sequence types (STs) identified, the most common were ST307 (15%), ST5241 (12%), ST15 (~9%), and ST25 (~6%). ST5241, 1 of 10 novel STs detected, is a single locus variant of ST636 carrying *dfrA14*, *tetD*, *qnrS*, and *oqxAB* resistance genes. The extended-spectrum β-lactamase (ESBL) gene *bla*_CTX_M-15_ was seen in 72% of *K. pneumoniae* genomes, while 8% encoded a carbapenemase. No isolate carried a combination of carbapenemase-producing genes. Four likely outbreak clusters from 1 facility, within STs 17, 25, 307, and 5241, were ESBL but not carbapenemase-bearing clones.

**Conclusions:**

This study uncovered known and novel *K. pneumoniae* lineages circulating in 3 hospitals in Southwest Nigeria that include multidrug-resistant ESBL producers. Carbapenemase-producing isolates remain uncommon. WGS retrospectively identified outbreak clusters, pointing to the value of genomic approaches in AMR surveillance for improving infection prevention and control in Nigerian hospitals.

*Klebsiella* is a ubiquitous gram-negative genus that can cause a variety of opportunistic infections [1, 2]. *Klebsiella pneumoniae,* the species most commonly associated with human disease, is frequently implicated in life-threatening bacteremia and sepsis arising from translocations from nonsterile niches or medical devices in hospitals [3]. Although the precise burden of infectious diseases resulting from *Klebsiella* spp. is unknown in Africa, there is an upward trend in reports of *K. pneumoniae*–associated bloodstream infections, including from Nigeria, Côte d’Ivoire, Malawi, Gambia, and South Africa [4–8]. There have also been isolated case reports of non-*pneumoniae* infections in Nigeria [9]. Altogether, these infections have high mortality rates and incur high costs [10, 11].

Rigorous surveillance of *Klebsiella* clones circulating within Nigerian hospitals and communities is needed to inform treatment guidelines and prioritize antimicrobial-resistance (AMR) interventions. Whole-genome sequencing (WGS) offers the attractive prospect of meeting this need within local resource constraints. WGS tools also provide taxonomic resolution below serotype levels, a feat impossible in resource-limited settings by classical typing methods [12]. Mapping the diversity of *Klebsiella* populations at high resolution, with spatiotemporal dynamics, also makes it possible to elucidate the origin and dissemination of AMR [12, 13].

In contrast to similarly constrained settings elsewhere in Africa [14–16], few Nigeria reports include in-depth analysis of more than a handful of *Klebsiella* isolates. For example, right ahead of this study, Pathogenwatch, which sources its public genomes from the European Nucleotide Archive (ENA), included only 109 *Klebsiella* genomes from Nigeria [17]. Nigeria recently initiated national AMR surveillance following commissioning of a 2017 National Action Plan, and the Nigeria Centre for Disease Control (NCDC) enrolled the country in the World Health Organization’s Global Antimicrobial Resistance Surveillance System (GLASS) [18]. The NCDC is building a network that currently consists of 9 AMR sentinel hospital laboratories and 2 national reference laboratories [19]. The very low number of *Klebsiella* genomes from Nigeria compelled the Nigeria node of the Global Health Research Unit (GHRU) on Genomic Surveillance of AMR, which provides WGS services to the reference laboratories, to collect and sequence retrospective isolates. In addition to increasing the number of relevant genomes in publicly available databases that can support prospective surveillance, this study aimed to understand the population structure, evolution, pathogenicity, and transmission dynamics of this critical-priority pathogen circulating in southwestern Nigeria.

## METHODS

### Ethical Considerations

Most genomes included in this study were acquired from surveillance without linked patient data. We additionally integrated data from the Obafemi Awolowo University (OAU) Teaching Hospital sentinel site, as approved by the Ethics and Research Committee, Obafemi Awolowo University Teaching Hospitals Complex, with registration number ERC/2017/05/06.

### GHRU Nigeria Workflow

#### Strains, Reidentification, and Antimicrobial Susceptibility Testing

The NCDC, Nigeria’s AMR surveillance coordinating center, requested from the network cryopreserved invasive isolates (from blood, cerebrospinal fluid, and urine) from 2016 to 2018 to develop a local genome database. Sentinel laboratories provided the national reference laboratory with strains and information on species identity, clinical diagnosis, and antimicrobial susceptibility tests (ASTs) via WHONET [20]. At the national reference laboratory, in collaboration with the GHRU, isolate identification and ASTs were validated using the VITEK system (version 2.0) https://www.biomerieux-usa.com/vitek-2, testing, as appropriate, amikacin, gentamicin, ampicillin, amoxicillin/clavulanic acid, piperacillin/tazobactam, cefuroxime, cefuroxime-axetil, cefepime, ceftriaxone, cefoperazone/sulbactam, nitrofurantoin, nalidixic acid, ciprofloxacin, ertapenem, imipenem, meropenem, trimethoprim-sulfamethoxazole, colistin, and tigecycline [21]. Antimicrobial susceptibility test results were interpreted in line with the CLSI (Clinical and Laboratory Standards Institute) standards [22].

#### DNA Extraction, Library Preparation, and Sequencing

Genomic DNA was extracted using the Wizard DNA extraction kit (Promega; catalog no. A1125). DNA was quantified using a dsDNA Broad Range fluorometric quantification assay (Invitrogen; catalog no. Q32853). Double-stranded DNA libraries (average, 500 bp) were prepared using the Covaris LC220 for fragmentation and NEBNext Ultra II FS DNA library kit for Illumina with 384-unique indexes (New England Biolabs; catalog no. E6617L). Libraries were sequenced using the HiSeq X10 with 150 bp paired-end chemistry (Illumina).

#### Genome Assembly

Genome assembly was carried out according to the GHRU protocol (https://www.protocols.io/view/ghru-genomic-surveillance-of-antimicrobial-resista-bpn6mmhe). Parameters for post-assembly quality checks include the total genome size (between 4 584 497 bp to 7 012 008 bp), N50 score (>25 000), contaminant level (<5%), and number of contigs (< 300). Only *Klebsiella* genomes that passed all quality checks (*K. pneumoniae*, n = 134; *Klebsiella quasipneumoniae*, n = 5) were selected for downstream analysis.

### Single Nucleotide Polymorphism Analysis

Sequence reads were mapped to the reference genome of the *K. pneumoniae* strain NTUH-K2044 (Genbank Accession number GCF_009497695.1) to determine evolutionary relationships among the isolates according to GHRU protocol (https://www.protocols.io/view/ghru-genomic-surveillance-of-antimicrobial-resista-bpn6mmhe). Pairwise single nucleotide polymorphism (SNP) distances for likely outbreak isolates from OAU were calculated from the pseudo-genome alignment using FastaDist (https://gitlab.com/antunderwood/fastadist). Outbreak isolates belonging to sequence types (STs) 17, 25, and 307 were aligned to reference genomes NZ_CP009461.1, NZ_CP031810.1, and NZ_CP022924.1 respectively, selected with Bactinspector (https://gitlab.com/antunderwood/bactinspector/). The ST636 reference EuSCAPE_UK136 was selected from Pathogenwatch collections, being the only known single locus variant of ST5241.

### Prediction of AMR Determinants, Virulence Factors, and Plasmid Profiling

The AMR determinants and plasmid replicons were predicted in accordance with the aforementioned GHRU protocol. *Omp* mutations, virulence score, and acquired virulence genes were determined using Kleborate (version 2.0.0; https://github.com/katholt/Kleborate) [23, 24].

### Concordance Analysis

Concordance between phenotypic and genotypic AMR for aminoglycosides, β-lactams, carbapenem, cephalosporin, quinolone, and trimethoprim was determined by using GitLab (https://gitlab.com/-/snippets/2050300) based on a protocol (https://glcdn.githack.com/cgps/ghru/ghrur/raw/master/docs/articles/simple_concordance_example.html) that uses the epi.tests function of the epiR package to calculate sensitivity and specificity with confidence bounds.

### Identification of Multilocus Sequence Types

Multilocus STs (MLSTs) according to the Pasteur scheme were determined by following the aforementioned GHRU protocol (https://www.protocols.io/view/ghru-genomic-surveillance-of-antimicrobial-resista-bpn6mmhe) [25]. We also used Kleborate (version2.0.0; https://github.com/katholt/Kleborate) to identify the ST most closely related to novel STs (in terms of locus variants).

### Availability of Sequence Data

Raw sequence datasets generated during this study have been deposited in the ENA with bioproject number PRJEB29739 (https://www.ebi.ac.uk/ena/browser/view/PRJEB29739). Accessions are available in Supplementary Table 3.

## RESULTS

### Epidemiology, Sequence Types, Virulence-Associated Determinants, and AMR Determinants of *Klebsiella* From Southwestern Nigeria Hospitals

Retrospective *Klebsiella* isolates were collected from 3 hospitals that had archival isolates: OAU Teaching Hospital (Ile-Ife, Nigeria; n = 92), University College Hospital (UCH; Ibadan, Nigeria; n = 30), and Ladoke Akintola University Teaching Hospital (LAU; Oshogbo, Nigeria; n = 12). Of the 134 isolates, 87 were from blood, comprising 45 from OAU, 30 from UCH, and 12 from LAU. We additionally sequenced 47 nonblood isolates from OAU, recovered from urine (n = 37), ocular (n = 2), stool (1), throat (n = 2), and rectal (n = 5) swabs.

Of the 134 genomes confirmed as *K. pneumoniae* by WGS, 33 (25%) were sent from the sentinels as either *Acinetobacter baumannii*, *Pseudomonas aeruginosa*, or other Enterobacteriales (Supplementary Table 1). Prior to sequencing, 18 (13%) were identified by the reference laboratory VITEK as other Enterobacteriales, with novel and uncommon *Klebsiella* STs later found to be predominant among those misidentified. Additionally, all WGS-identified *K. quasipneumoniae* isolates were identified either as *K. pneumoniae* or *Enterobacter* complexes by VITEK (Supplementary Table 1). The phylogenetic tree, epidemiological data, and analyses data can be visualized in Microreact for retrospective *K. pneumoniae* (https://microreact.org/project/GHRUNigeriaKpneumoniae/2a856694) and OAU outbreak isolates (ST17: https://microreact.org/project/brfq17BXzwmqptfcrNRfZR/71b2f0f1; ST25: https://microreact.org/project/8FP4F1D5fSQMv6FDxbR39b/bd381f0b; ST307: https://microreact.org/project/sV5NsJ8szcorvFAeFQgV4E/6498edf1; ST5241: https://microreact.org/project/u5RPX7CjyitjRYMQ9ByW4W/1a3570e2).

**Figure 1.**
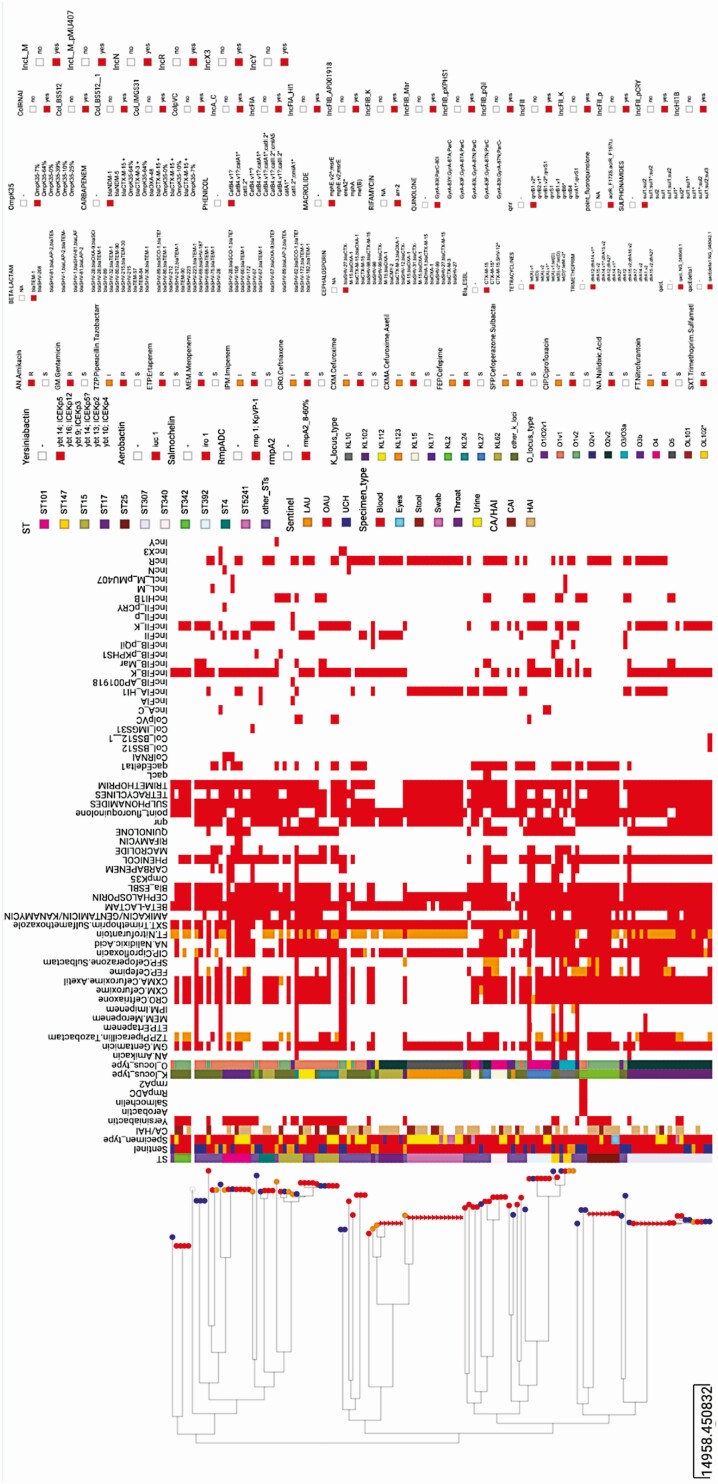
Epidemiological data, virulence determinants, antibiotic profile (phenotypic resistance), and AMR determinants in *Klebsiella pneumoniae* genomes ordered by phylogeny. The heat map shows the presence (red) or absence (blank) of virulence determinants, phenotypic resistance, AMR genes, and plasmid replicon genes. Tree nodes represent the origin of isolate collection. The shape of the tree nodes depicts the outbreak (star) and non-outbreak (circle) isolates. The data are available at https://microreact.org/project/GHRUNigeriaKpneumoniae/2a856694. Abbreviation: AMR, antimicrobial resistance.

Overall, the *K. pneumoniae* isolates sequenced in this study belonged to 39 different STs, with ST307, ST5241, ST15, and ST25 being the most common (Figure 1). Only 4 STs (ST15, ST101, ST147, and ST307) were found across the 3 sentinel sites (Figure 1). *Klebsiella quasipneumoniae* genomes (n = 5) belonged to 4 STs: ST1602, ST2133, ST5250 (n = 1 each), and ST5249 (n = 2).

We identified few virulence genes associated with invasiveness, including yersiniabactin (*ybt* genes and *fyuA*; n = 42, 31.34%), aerobactin (*iuc*; n = 2), salmochelin (*iro*; n = 2), and capsule expression upregulators *rmpADC* and *rmpA2* (n = 2). Only 2 ST86 isolates had a virulence score of 3, the highest recorded in our collection. No acquired *K. pneumoniae* virulence gene was detected in the novel ST5241(Figure 1) or in the non-*pneumoniae Klebsiella* species.

We detected 33 different phenotypically defined K (capsular) loci from the invasive *K. pneumoniae* isolates sequenced, with KL102, KL123, KL2, KL62, and KL27 representing the 5 most common K loci (Supplementary Table 2). Eleven different O loci were detected among the invasive *K. pneumoniae* isolates recovered from bloodstream infections. Collectively, the 3 O loci—O1v1, O2v2, and O1v2—accounted for more than 65% of the *K. pneumoniae* strains, and O5 was solely detected in ST5241 genomes.

Genes conferring resistance or reduced susceptibility to at least 5 antibiotic classes were detected in 116 (86.6%) *K. pneumoniae* genomes. Aside from core *bla*_*SHV*_, these included 8 β-lactamase genes, of which the extended-spectrum β-lactamase (ESBL) *bla*_CTX-M-15_ was by far the most common, present in 71.6% (n = 96) of the *K. pneumoniae* isolates belonging to 33 STs (Figure 1). As seen in other studies, we found this ESBL in strains with plasmids belonging to IncFIB(K) (n = 70/96), IncFII(K) (n = 51/96), and IncR (n = 43/96) incompatibility groups [26, 27]. The *bla*_NDM-1_ (n = 8/134), *bla*_NDM-5_ (n = 2/134), and *bla*_OXA-48_ (n = 1/134) genes were the only carbapenemases detected, and these occurred in isolates belonging to 8 STs, including ST392 (*bla*_NDM-1_; n = 2); ST147/147-1-locus-variant (*bla*_NDM-1_; n = 2); ST530 (*bla*_NDM-5_; n = 2); ST43, ST15, ST307, and ST716 (*bla*_NDM-1_; n = 1 each); and ST219 (*bla*_OXA-48_; n = 1) (Figure 1). None of the isolates carried a combination of carbapenemase-producing genes. OmpK35 truncations were observed together with *bla*_CTX-M-15_ in 11 isolates and with *bla*_NDM-1_ in 5 isolates. Altogether, 18 (13.5%) *K. pneumoniae* strains carried carbapenemase genes and/or ESBL genes plus porin defects known to confer carbapenem resistance [28]. Phenotypic antimicrobial susceptibility testing found 11 of 128 isolates carbapenem nonsusceptible, and *bla*_NDM-1_ (n = 6), *bla*_NDM-5_ (n = 2), and/or *bla*_CTX-M-15_ and OmpK35 truncations (n = 1) could account for nonsusceptibility in 9 of them (Figure 1). Concordance analysis showed that phenotypic AST and genotypic AMR gene prediction results agree substantially for carbapenems (concordance: 92.2%; sensitivity: 82% [48.22– 97.72%]; specificity: 93.2% [86.97–97%]). As we did not particularly aim to sample carbapenem-resistant *K. pneumoniae*, this might have been partially responsible for the low rate of carbapenem resistance observed in our study compared with other studies in this supplement.

Reduced susceptibility to nalidixic acid (n = 70/125) or ciprofloxacin (n = 114/125) could be explained by the presence of 1 or more combinations of mutations in the quinolone resistance determining region (QRDR) of *gyrA* and *parC* (n = 60), plasmid-mediated quinolone resistance genes (*qnrS*, *qnrA*, *qnrB*; n = 82), and/or efflux-mediating mutations in the *acrR* gene (n = 98) (Figure 1).

The 5 *K. quasipneumoniae* were resistant to phenicols, tetracyclines, sulfonamides, trimethoprim, and β-lactams (https://microreact.org/project/4f6Y56ECE979wkj8oDBhk2/dee16dac). One LAU isolate, collected 6 months after an outbreak of *K. quasipneumoniae* strains carrying *bla*_*NDM-5*_ in Abuja, Nigeria, was remotely related to those outbreak strains (SNP distance ≥235), but, like the other 4 *K. quasipneumoniae* from the current study, it lacked carbapenemase genes [9].

### Retrospective Characterization of Possible Outbreaks

**Figure 2.**
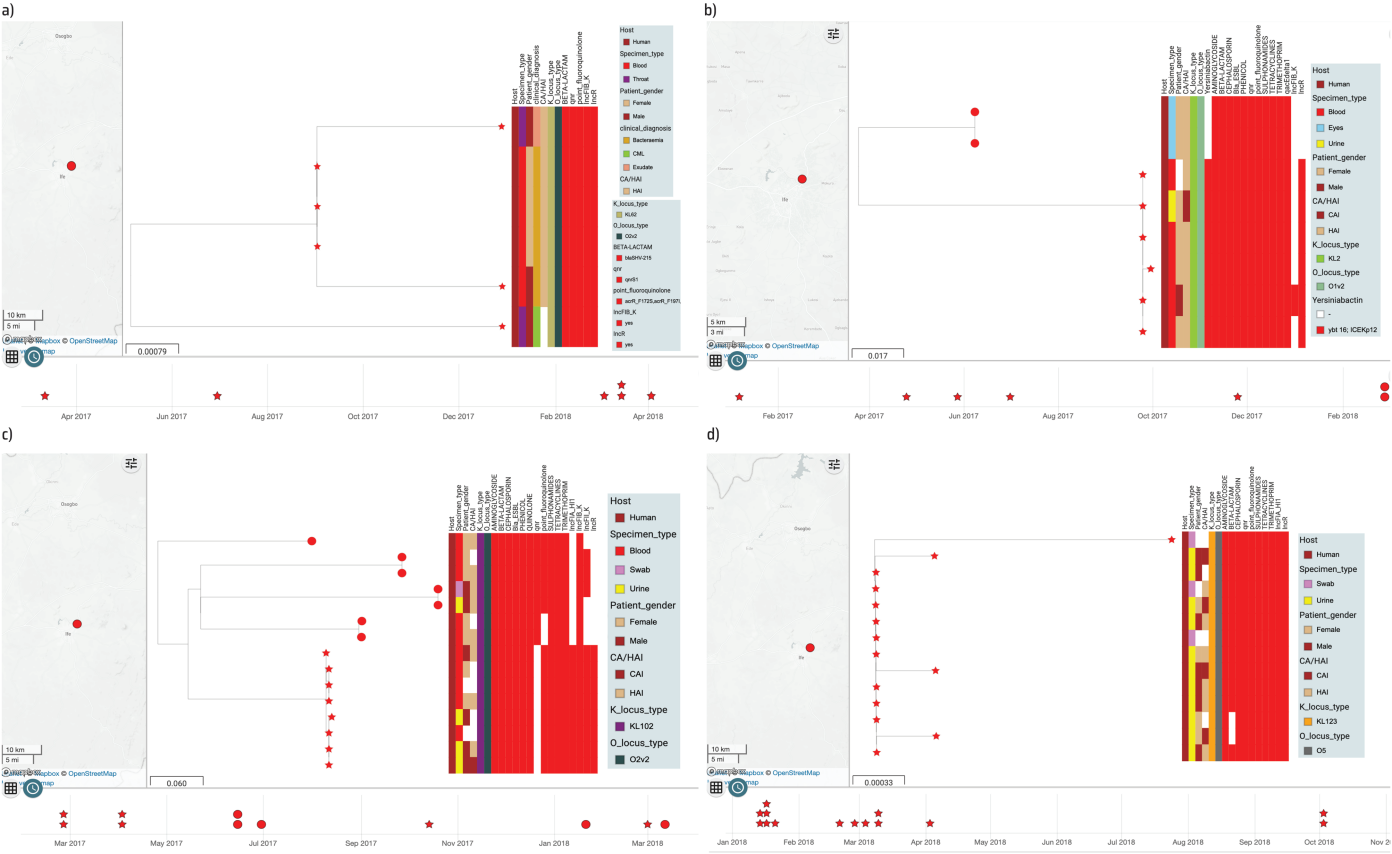
The timeline of likely outbreak of *Klebsiella pneumoniae* clones belonging to STs 17 (*a*), 25 (*b*), 307(*c*), and 5241(*d*) from the OAU sentinel site. The shape of the tree nodes depicts the outbreak (star) and non-outbreak (circle) isolate, while the color of the tree nodes depicts the sentinel site. The data are available at https://microreact.org/project/brfq17BXzwmqptfcrNRfZR/71b2f0f1 (ST17), https://microreact.org/project/8FP4F1D5fSQMv6FDxbR39b/bd381f0b (ST25), https://microreact.org/project/sV5NsJ8szcorvFAeFQgV4E/6498edf1 (ST307), and https://microreact.org/project/u5RPX7CjyitjRYMQ9ByW4W/1a3570e2 (ST5241). Abbreviations: OAU, Obafemi Awolowo University; ST, sequence type.

Recovery dates for 74 of 92 isolates from the OAU facility were supplied. In under 1 year, between 6 and 14 isolates were recovered belonging to ST17, ST25, ST307, and ST5241. We noticed clusters of closely related isolates that were recovered within short periods, and we hypothesized that these might represent unreported/undetected outbreaks. Intracluster pairwise distances within the potential outbreak clades ranged from 1 to 3 SNPs, and intercluster pairwise distances within the same ST ranged from 46 to 370. Temporally clustered, closely related clonal groups within each of the 4 STs (Figure 2), with similar AMR gene, virulence gene, and plasmid replicon profiles, support that these clusters represent outbreaks, and that nosocomial transmission contributes heavily to invasive *Klebsiella* infections. The outbreaks mostly involved ESBL lineages but no carbapenemase producers.

## Discussion

This report on the population structure of *Klebsiella* in 3 tertiary hospitals addresses critical knowledge gaps regarding the characteristics of *K. pneumoniae* in Nigeria and presents 5 *K. quasipneumoniae* genomes, which without WGS cannot be differentiated from *K. pneumoniae* in our setting, even at the reference laboratory level. Although the VITEK2 identification system is innovative and is currently used in reference microbiology laboratories worldwide, the accuracy of its identification is dependent on the isolate’s phenotypic variability (eg, variation in colony morphology) and the completeness of the system’s identification database [29, 30]. The misidentification of *K. quasipneumoniae* and *Klebsiella variicola* as *K. pneumoniae* has been reported previously, and thus is not unique to our setting [30]. Ours and other data reveal that multiple *K. quasipneumoniae* lineages, including resistant ones, circulate in Nigeria [9]. We also corroborate previous reports on the diversity of *K. pneumoniae*, which pose challenges as well as opportunities for curtailing and combating this pathogen [31, 32].

The high-prevalence genes conferring resistance to fluoroquinolones and extended-spectrum β-lactams, last-line options for Nigeria’s least-affluent patients, in the most common lineages, reported here is of concern despite relatively low rates of carbapenem resistance in our setting, or elsewhere in Africa, compared with other countries [15, 33–35]. Moreover, while uncommon in this study, ST147 and ST392 strains bearing *bla*_NDM-1_, and ST101 strains carrying *bla*_OXA-48_ were documented [36]. (Multiple resistant isolates that co-carry *bla*_NDM-1_ and *bla*_OXA-48_ have been observed in other parts of Africa [Morocco, Tunisia, Egypt], Asia [Iran, Pakistan, Turkey], and Europe, within the last decade.) These and other carbapenem-resistant lineages reported in other papers in this supplement issue signify evolution, spread, and persistence of worrisome high-risk lineages [37, 38]). The predominant lineages presented in the current report deserve close and careful monitoring in national prospective surveillance, and further work is necessary to identify and track mobile elements they carry. The preponderance of plasmids in the *Klebsiella* strains sequenced reflects the known propensity of this genus to harbor and diversify mobile elements in the face of high selective pressure and has implications for resistance in the many species to which *K. pneumoniae* transmits DNA [2, 39].

Clonal group (CG) 307 is believed to be displacing the notorious CG258 as a primary lineage in South Africa, Italy, Colombia, and the United States, and it has been suggested that it may be more virulent [33, 36, 40–43]. The high ST307 frequency here, as in in Malawi and South Africa, may explain the low overall prevalence of carbapenem resistance we observed compared with other locations [14, 17, 34, 44]. We recorded 21 ST307 isolates, but no ST258 isolates, and only 7 CG258 isolates: 5 ST340 and 2 ST11.

Clusters of likely clonal isolates within clonal complexes 15, 25, 17, ST5241, 392, and 101 were identified among retrospective isolates from OAU, the most sampled sentinel site; within clonal complexes 15, 25, and 17, as well as ST307, ST5241, ST392, and ST101. The close genetic distances observed within 4 of these clones (<4 pairwise SNP differences in each case) and the similar or identical resistance, virulence, and plasmid replicon profiles, and temporal clustering of their isolation, strongly suggest that each of these clusters represents a retrospectively identified outbreak. As fewer retrospective isolates were stored at the other 2 institutions, we cannot rule out similar occurrences that are below our limits of detection. Outbreaks were caused by highly drug-resistant lineages such as the ST17 cluster, but also more sensitive ones like the ST5241 and ST307 *K. pneumoniae* clusters. While outbreaks can sometimes be detected by phenotypic testing in diagnostic laboratories alone, the minimal biochemical and narrow disc diffusion test repertoires used at sentinel laboratories, which underlie many species’ misidentifications (Supplementary Table 1), make detection unlikely when the number of outbreak isolates is small. Our data show that, as in countries with more intensive surveillance, outbreaks likely commonly occur within health facilities in Nigeria and elsewhere in West Africa and will typically be missed without genomic support [7, 12, 45, 46]. The OAU and UCH currently provide subsidized blood culture for many patients, but most Nigerian facilities with blood culture do not, and health insurance coverage is low. Therefore, identifying outbreaks is likely to be curtailed by patients’ inability to pay. Our data reveal that recent efforts to strengthen Nigeria’s AMR response and boost infection prevention and control (IPC) could be mutually enhancing if blood culture of routine febrile patient blood culture is facility-supported, and if prospective genomic surveillance is used.

This study has some limitations. First, very few archival isolates from only 3 facilities were available, and epidemiological data were incomplete. Furthermore, the STs observed here were sampled only from invasive infections. Nonetheless, this study represents an important starting point for documenting *Klebsiella* lineages, and we will build on these findings with prospective surveillance at more hospitals in southwestern Nigeria, which have now been enrolled in the national surveillance system.

### Conclusions

We have detailed the characteristics of *Klebsiella* isolates from 3 southwestern hospitals in Nigeria. In addition to incorporating Nigeria-derived data into global phylogeographies of multidrug-resistant *K. pneumoniae*, our findings point to the significance of ST307 in Nigeria. Carbapenem-resistant STs 147 and 392, as well as the novel ST5241 *K. pneumoniae*, may also represent future surveillance priorities for southwestern Nigeria. We have shown the benefits of public health genomic surveillance of pathogens––in particular, the potential for outbreak identification. Infection prevention and control is a key pillar of Nigeria’s AMR National Action Plan and the data from this paper suggest that genomic surveillance and IPC implementation could be mutually reinforcing in this collaboration among scientists, health institutions, and the public health authority in Nigeria.

## Supplementary Data

Supplementary materials are available at *Clinical Infectious Diseases* online. Consisting of data provided by the authors to benefit the reader, the posted materials are not copyedited and are the sole responsibility of the authors, so questions or comments should be addressed to the corresponding author.

ciab769_suppl_Supplementary_Material

ciab769_suppl_Supplementary_Table_1

ciab769_suppl_Supplementary_Table_2

ciab769_suppl_Supplementary_Table_3

## Notes

We sincerely appreciate technical assistance from sentinel site staff as well as Ifeoluwa Akintayo, Ifeoluwa Komolafe, and Faith Ifeoluwa Popoola. We also thank the team of curators of the Institut Pasteur MLST/cgMLST databases for curating the data and making them publicly available at https://bigsdb.pasteur.fr. We thank Lucy Goodchild for editorial assistance.

***Supplement sponsorship.*** The supplement is sponsored by the UK National Institute for Health Research Global Health Research Unit on Genomic Surveillance of AMR.

***Disclaimer.*** The funders had no role in the content, crafting, or submission of this paper. The views expressed in this publication are those of the authors and not necessarily those of the funders or their affiliates.

***Financial support.*** This work was supported by Official Development Assistance (ODA) funding from the National Institute for Health Research (grant number 16/136/111: NIHR Global Health Research Unit on Genomic Surveillance of Antimicrobial Resistance).

This research was commissioned by the National Institute for Health Research using ODA funding. I. N. O. is an African Research Leader supported by the UK Medical Research Council (MRC) and the UK Department for International Development (DFID) under the MRC/DFID Concordat agreement that is also part of the EDCTP2 program supported by the European Union. The views expressed in this publication are those of the authors and not necessarily those of the funders. I. N. O. reports grant and nonfinancial support from International Vaccine Institute (SETA Award to University of Ibadan), and is a member of the Bill & Melinda Gates Foundation (BMGF) Surveillance Advisors Group (SAG) (from 2019–current). M. K. reports grands or funding from Centre for Genomic Pathogen Surveillance (CGPS) during the conduct of the study. M. A. reports grants or funding from Wellcome Connecting Science during the conduct of the study.

***Potential conflicts of interest.*** M. K. reports grants or contracts, support for attending meetings and/or travel, and receipt of equipment, materials, drugs, medical writing, gifts, or other services from the National Institute for Health Research (NIHR) outside of the submitted work. No other authors reported conflicts of interest. All authors have submitted the ICMJE Form for Disclosure of Potential Conflicts of Interest. Conflicts that the editors consider relevant to the content of the manuscript have been disclosed.

***Acknowledgments.*** Members of the NIHR Global Health Research Unit on Genomic Surveillance of Antimicrobial Resistance: Khalil Abudahab, Harry Harste, Dawn Muddyman, Ben Taylor, and Nicole Wheeler of the Centre for Genomic Pathogen Surveillance, Big Data Institute, University of Oxford, Oxford, United Kingdom, and Wellcome Genome Campus, Hinxton, United Kingdom; Pilar Donado-Godoy, Johan Fabian Bernal, Alejandra Arevalo, Maria Fernanda Valencia, and Erik C. D. Osma Castro of the Colombian Integrated Program for Antimicrobial Resistance Surveillance—Coipars, Centro de Investigación Tibaitatá, Corporación Colombiana de Investigación Agropecuaria (AGROSAVIA), Tibaitatá–Mosquera, Cundinamarca, Colombia; K. L. Ravikumar, Geetha Nagaraj, Varun Shamanna, Vandana Govindan, Akshata Prabhu, D. Sravani, M. R. Shincy, Steffimole Rose, and K. N. Ravishankar of the Central Research Laboratory, Kempegowda Institute of Medical Sciences, Bengaluru, India; Jolaade J. Ajiboye of the Department of Pharmaceutical Microbiology, Faculty of Pharmacy, University of Ibadan, Nigeria; Celia Carlos, Marietta L. Lagrada, Polle Krystle V. Macaranas, Agnettah M. Olorosa, June M. Gayeta, and Elmer M. Herrera of the Antimicrobial Resistance Surveillance Reference Laboratory, Research Institute for Tropical Medicine, Muntinlupa, The Philippines; Ali Molloy (alimolloy.com); John Stelling, The Brigham and Women’s Hospital; and Carolin Vegvari, Imperial College London.
