# Supplementary material for: Clones and Clusters of Antimicrobial-Resistant Klebsiella From Southwestern Nigeria
Source: Clin Infect Dis. 2021 Dec 1;73(Suppl 4):S308–15. doi: 10.1093/cid/ciab769 (PMC8634535; doi:10.1093/cid/ciab769)
Supplement: ciab769_suppl_Supplementary_Material [file ciab769_suppl_supplementary_material.docx]

**Supplementary Information**

**Clones and clusters of antimicrobial-resistant *Klebsiella* from southwestern Nigeria**

Ayorinde O. Afolayan^1^, Anderson O. Oaikhena^1^. Aaron O. Aboderin^2^, Olatunde F. Olabisi^2^, Adewale A. Amupitan^2^, Oyekola V. Abiri^2^, Veronica O. Ogunleye^3^, Anthony Underwood^5^, Erkison Ewomazino Odih^1^, Abolaji T. Adeyemo^4^, Adeyemi T. Adeyemo^2^, Temitope O. Obadare^2^, Sophia David^5^, Silvia Argimón^5^, Monica Abrudan^5^, Abiodun Egwuenu^6^, Chikwe Ihekweazu^6^, David M. Aanensen^5^, Iruka N. Okeke^1^ and the NIHR Global Health Research Unit (GHRU) on Genomic Surveillance of Antimicrobial Resistance ^a^

^1^Global Health Research Unit on Genomic Surveillance of Antimicrobial Resistance, Department of Pharmaceutical Microbiology, Faculty of Pharmacy, University of Ibadan, Oyo State, Nigeria.

^2^Department of Medical Microbiology and Parasitology, Obafemi Awolowo University, Ile-Ife, Osun State, Nigeria.

^3^University College Hospital, Ibadan, Oyo State, Nigeria.

^4^Department of Medical Microbiology and Parasitology, LAUTECH Teaching Hospital, Osogbo, Nigeria. LAUTECH Teaching Hospital, Osogbo, Nigeria

^5^Centre for Genomic Pathogen Surveillance, Big Data Institute, University of Oxford, Old Road Campus, Oxford, United Kingdom and Wellcome Genome Campus, Hinxton, UK

^6^Nigeria Centre for Disease Control, Jabi, Abuja, Nigeria.

^a^ Members of the NIHR Global Health Research Unit on Genomic Surveillance of Antimicrobial Resistance are listed in the Acknowledgments.

**SUPPLEMENTARY TABLES**

**Supplementary Table 1.** Accession numbers of *Klebsiella* genomes.

[Available in Excel spreadsheet]

**Supplementary Table 2.** Epidemiological data, virulence determinants, antibiotic profile (phenotypic resistance), and antimicrobial resistance determinants in *K. pneumoniae* genomes.

[Available in Excel spreadsheet]
